# Novel systemic therapies in atopic dermatitis: what do we need to fulfil the promise of a treatment revolution?

**DOI:** 10.12688/f1000research.17039.1

**Published:** 2019-01-31

**Authors:** Helen Alexander, Thomas Patton, Zarif K. Jabbar-Lopez, Andrea Manca, Carsten Flohr

**Affiliations:** 1 Unit for Population-Based Dermatology Research, St John's Institute of Dermatology, Guy's and St Thomas' NHS Foundation Trust and King's College London, London, UK; 2 Centre for Health Economics, University of York, York, UK

**Keywords:** Atopic dermatitis, eczema

## Abstract

Patients with atopic dermatitis (AD) who do not adequately respond to topical therapy and phototherapy often need systemic immunomodulatory treatment to control their symptoms. Conventional systemic agents, such as ciclosporin, azathioprine, and methotrexate, have been used for decades, but there are concerns about their safety profile. There are now many novel systemic agents emerging through clinical trials, which may have great potential in the treatment of AD. Despite this, there are very few data comparing the performance of these drugs against each other. The purpose of this article is to review the current systemic therapies in AD and present an indirect comparison of systemic AD treatments using effectiveness and safety data from published randomised controlled trials, highlighting important remaining gaps in knowledge. Although the latest developments in systemic AD treatments are exciting and dearly needed, further work is required before the promise of a therapeutic revolution becomes reality.

## Introduction

Atopic dermatitis (AD) affects 15–30% of children and 5% of adults and carries profound functional, psychological, and social morbidity ^1– 3^. Although mild and moderate AD can usually be managed with topical treatments and/or phototherapy, approximately 2% of people with AD require systemic treatments to induce adequate symptom control ^4, 5^.

We are entering an exciting era, described by some as a therapeutic revolution ^6– 8^, as dozens of novel systemic treatments are being developed for AD. These targeted biologic and small molecule agents and the conventional systemic immunosuppressive AD treatments provide an increasingly broad range of therapeutic options. Although there is some clinical guidance on when to start systemic therapy, many evidence gaps remain regarding the comparative performance of these drugs ^9, 10^. Moreover, the lack of a gold standard conventional systemic therapy for AD means there is no benchmark against which to compare the performance of novel agents. Many factors, including the impact on disease severity and quality of life as well as adverse events (AEs) and cost-effectiveness, play into the complex treatment decision-making process when clinicians and patients agree on choosing a particular therapy and in the formulation of treatment guidance produced by stakeholders, such as the UK National Institute for Health and Care Excellence (NICE).

Based on a systematic search of the literature, we discuss the latest developments in systemic AD treatments. We present an indirect comparison of novel and conventional systemic AD treatments with regard to treatment efficacy, safety, and cost-effectiveness and highlight important gaps that need to be filled.

## Conventional systemic atopic dermatitis treatments

The main conventional systemic treatments for AD are ciclosporin, methotrexate, and azathioprine ^11– 13^. Mycophenolate mofetil is less commonly used. Some of these agents have been used to treat severe AD for decades despite a lack of robust randomised controlled trial (RCT) evidence. Ciclosporin is a calcineurin inhibitor that inhibits T-cell-dependent immune responses. It has a rapid onset of action with significant improvement in disease severity often seen within a few weeks. However, relapse is commonly seen after treatment withdrawal. Methotrexate is a folic acid antagonist, but its exact mechanism of action in inflammatory diseases, including AD, is not fully understood. It has a relatively slow onset of action, like azathioprine. The latter exerts its anti-inflammatory effects by inhibition of *de novo* purine synthesis leading to impaired leucocyte proliferation. Anecdotally, azathioprine and methotrexate have the potential to alter the natural history of the disease and induce long-term remission, although there is currently no RCT evidence to confirm this ^14– 16^. Mycophenolate mofetil blocks *de novo* guanine synthesis via the inhibition of inosine monophosphate dehydrogenase leading to impaired leucocyte proliferation. The safety profiles of azathioprine and ciclosporin in particular are of concern. Nephrotoxicity and hypertension are the most significant side effects of ciclosporin. As a result, the United States Food and Drug Administration recommends limiting its continuous use to one year in psoriasis patients ^17^. Azathioprine can cause myelosuppression and carries an increased risk of infection, lymphoma, and non-melanoma skin cancer ^18– 22^. Methotrexate and mycophenolate mofetil are considered relatively safe medications, but long-term data from AD cohorts are missing at present. In practice, even when a conventional agent is working well in AD, most clinicians feel that these agents cannot be used for years, particularly because of the long-term risk of malignancy. The development of novel agents, with improved long-term safety, is therefore essential.

## Novel systemic atopic dermatitis treatments

**Figure 1.**
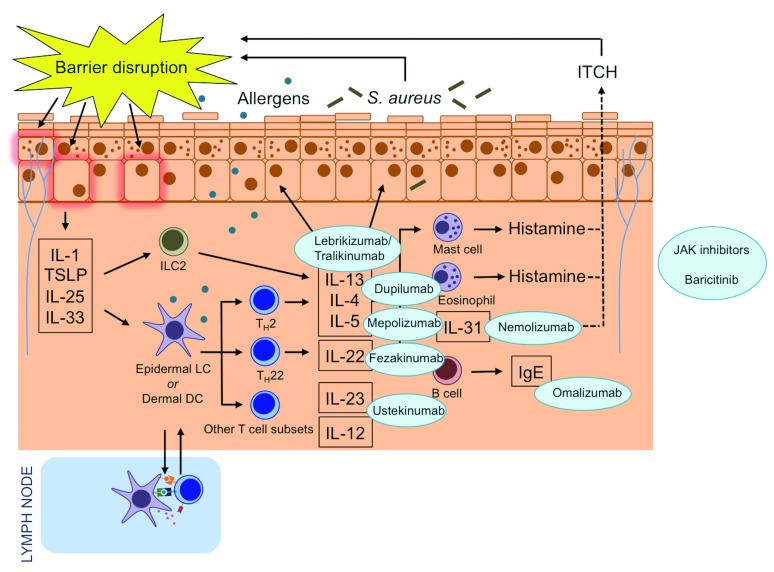
Atopic dermatitis pathogenesis and drug targets of novel systemic therapies. Novel systemic therapies target immune mediators in atopic dermatitis. Mepolizumab is a monoclonal antibody to interleukin-4 (IL-4). Omalizumab is a monoclonal anti-immunoglobulin E (IgE) antibody. Dupilumab is an IL-4 receptor α-antagonist that inhibits IL-4 and IL-13 signalling. Lebrikizumab and tralokinumab are monoclonal antibodies that bind to IL-13. Ustekinumab binds to the shared p40 subunit of IL-12 and IL-23 to regulate T helper type 1 (Th1) and Th17 pathways. Nemolizumab is a monoclonal antibody against IL-31 receptor A. Fezakinumab is an IL-22 antagonist. Baricitinib is a Janus kinase 1 (JAK1) and JAK2 inhibitor. DC, dendritic cell; ILC2, type 2 innate lymphoid cell; LC, Langerhans cell; S. aureus, *Staphylococcus aureus*; TSLP, thymic stromal lymphopoietin.

Thanks to our enhanced understanding of the complex immunological processes in AD skin, there are now many promising treatment targets ( Figure 1). Dupilumab is an interleukin (IL)-4 receptor α-antagonist that inhibits IL-4 and IL-13 signalling and has been approved in Europe and the United States for the treatment of adults with moderate-to-severe AD. Clinical trials are underway in children. In addition to dupilumab, the IL-13 inhibitors tralokinumab and lebrikizumab and the IL-31 receptor monoclonal antibody nemolizumab have also demonstrated good potential in clinical trials. Fezakinumab, a monoclonal antibody against IL-22, was effective in the treatment of patients with severe AD in a recent phase 2 trial. Janus kinase (JAK) inhibitors are used to treat a range of inflammatory diseases, and data demonstrating their efficacy in AD are now also emerging.

## Indirect comparison of atopic dermatitis systemic treatments

As there is a paucity of direct head-to-head trial data comparing treatments in AD, we have indirectly compared the performance of these drugs using data from published RCTs. In our analysis, we have included both blinded and open-label extension RCTs that were published up until 30 September 2018 and report efficacy and safety data of one or more systemic immunomodulatory treatments for moderate-to-severe AD. We have included only the conventional systemic RCTs in which the most commonly used conventional treatments were tested: ciclosporin, methotrexate, azathioprine, and mycophenolate mofetil. We included only trials that used a validated severity measure, such as the Eczema Area and Severity Index (EASI), Scoring Atopic Dermatitis (SCORAD) index, the Six Area Six Sign Atopic Dermatitis (SASSAD) severity score, or the Patient Oriented Eczema Measure (POEM).

### Overview of included randomised controlled trials

**Figure 2.**
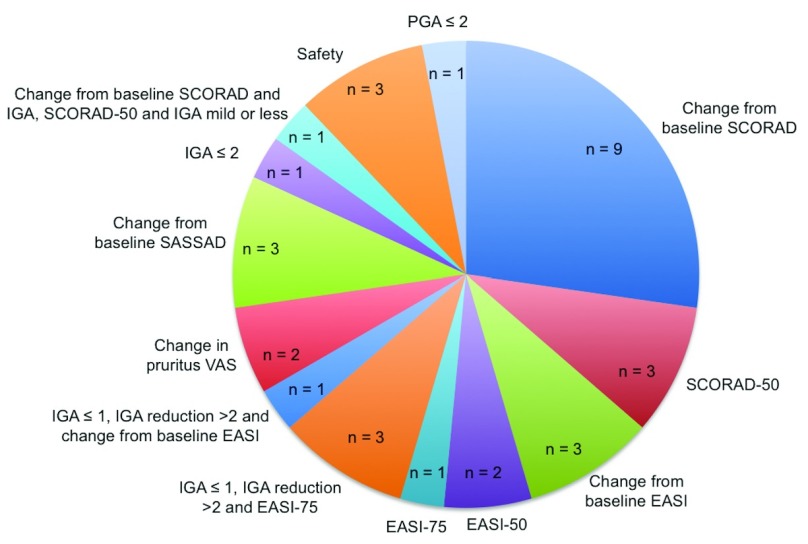
Primary end points of included trials. The diverse range of primary endpoints reported across included randomised controlled trials. EASI, Eczema Area and Severity Index; IGA, Investigator’s Global Assessment; PGA, Physician's Global Assessment; SASSAD, Six Area Six Sign Atopic Dermatitis; SCORAD, Scoring Atopic Dermatitis; VAS, visual analogue scale.

We included 33 trials ( Table 1). Most of the trials testing conventional systemic agents were small, and a single phase 3 dupilumab trial enrolled more patients than all the included conventional systemic trials ^23^. A total of 12 RCTs were head-to-head comparisons and 21 were placebo-controlled trials. The majority (85.7%, 12/14) of the conventional systemic agent trials were head-to-head comparisons, while there are currently no head-to-head trials including novel AD treatments. A diverse range of primary endpoints was used across these studies ( Figure 2).

**Table 1.** Summary of included randomised controlled trials.

Author	Year	Intervention	n	Study duration (weeks)
Harper *et al.* ^25^	2000	Ciclosporin burst versus continuous therapy	40	52
Granlund *et al.* ^26^	2001	Ciclosporin versus UVAB phototherapy	38	8
Pacor *et al.* ^27^	2004	Ciclosporin versus tacrolimus	30	8
Bemanian *et al.* ^28^	2005	Ciclosporin versus intravenous immunoglobulin	14	12
Schmitt *et al.* ^29^	2010	Ciclosporin versus prednisolone	38	6
El-Khalawany *et al.* ^30^	2013	Ciclosporin versus methotrexate	40	12
Koppelhus *et al.* ^31^	2014	Ciclosporin versus extracorporeal photopheresis	20	30
Jin *et al.* ^32^	2015	Ciclosporin versus ciclosporin + glucosamine	38	8
Kim *et al.* ^33^	2016	Ciclosporin versus ciclosporin + topical therapy	60	24
Goujon *et al.* ^34^	2017	Ciclosporin versus methotrexate	97	24
Berth-Jones *et al.* ^35^	2002	Azathioprine versus placebo	27	12
Meggitt *et al.* ^36^	2006	Azathioprine versus placebo	41	12
Schram *et al.* ^37^	2011	Azathioprine versus methotrexate	42	12
Gerbens *et al.* ^14^	2018	Azathioprine versus methotrexate	35	260
Oldhoff *et al.* ^38^	2005	Mepolizumab versus placebo	43	2
Iyengar *et al.* ^39^	2013	Omalizumab versus placebo	8	24
Beck *et al.* ^40^	2014	Dupilumab versus placebo	30	4
Beck *et al.* ^40^	2014	Dupilumab versus placebo	37	4
Beck *et al.* ^40^	2014	Dupilumab versus placebo	109	12
Beck *et al.* ^40^	2014	Dupilumab + topical glucocorticoids versus placebo	31	4
Thaçi *et al.* ^41^	2016	Dupilumab versus placebo	379	16
Simpson *et al.* ^23^	2016	Dupilumab versus placebo	671	16
Simpson *et al.* ^23^	2016	Dupilumab versus placebo	708	16
Blauvelt *et al.* ^42^	2017	Dupilumab versus placebo	740	52
de Bruin-Weller *et al.* ^43^	2018	Dupilumab versus placebo	390	16
Khattri *et al.* ^44^	2017	Ustekinumab versus placebo	33	33
Saeki *et al.* ^45^	2017	Ustekinumab versus placebo	79	12
Ruzicka *et al.* ^46^	2017	Nemolizumab versus placebo	264	12
Kabashima *et al.* ^47^	2018	Nemolizumab versus placebo	191	64
Wollenberg *et al.* ^48^	2018	Tralokinumab versus placebo	204	12
Guttman-Yassky *et al.* ^49^	2018	Fezakinumab versus placebo	60	10
Guttman-Yassky *et al.* ^50^	2018	Baricitinib versus placebo	124	16
Simpson *et al.* ^51^	2018	Lebrikizumab versus placebo	209	12

### Treatment effectiveness: physician-assessed severity measures

Different clinical severity score endpoints were reported across the trials ( Supplementary Figure 1). Many trials did not report the same scores, even as secondary or experimental endpoints, making indirect comparison of some treatments very difficult. In some cases, the scales of the same score differed across trials, thus further complicating clinical effectiveness comparisons. For instance, the Investigator’s Global Assessment (IGA) score was reported in 22 trials, but some used a five-point Likert scale while others used a six-point scale. Some trials reported the objective SCORAD score, whereas others published the full composite SCORAD index, which includes patient-reported pruritus and sleep disturbance.

**Figure 3.**
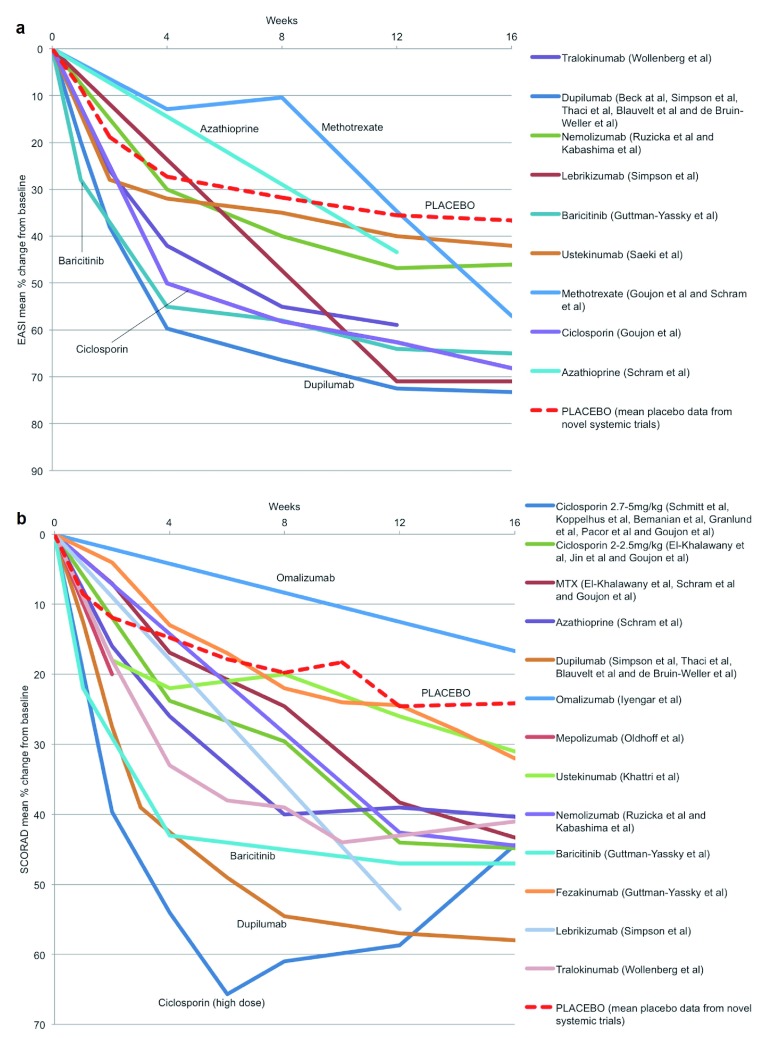
Treatment effectiveness: physician-assessed severity measures. Eczema Area and Severity Index (EASI) (a) and Scoring Atopic Dermatitis (SCORAD) (b) mean percentage change from baseline up to 16 weeks for systemic agents for which these data were reported. MTX, methotrexate.

We compared the mean percentage change from baseline for EASI and SCORAD for each of the systemic treatments where these data were available ( Figure 3a and 3b, Supplementary Figure 2a and 2b). We calculated a mean placebo response using pooled data from all novel systemic therapy RCTs. This indirect comparison suggests that ciclosporin at a dose of 2.7–5 mg/kg/day in the short-term and methotrexate and azathioprine in the longer term may perform as well as many of the novel systemic agents. The superior performance of dupilumab compared with ciclosporin (2.5–5 mg/kg/day) in terms of EASI is consistent with another indirect comparison in which logistic regression modelling was used to predict the EASI responses to these treatments ^24^. However, drug dosing is important, and an indirect comparison of effectiveness, as measured by SCORAD reduction induced by these agents, suggests that ciclosporin at a dose of 5 mg/kg/day may be as effective as dupilumab at least for short-term disease control.

### Treatment effectiveness: patient-reported severity measures

**Figure 4.**
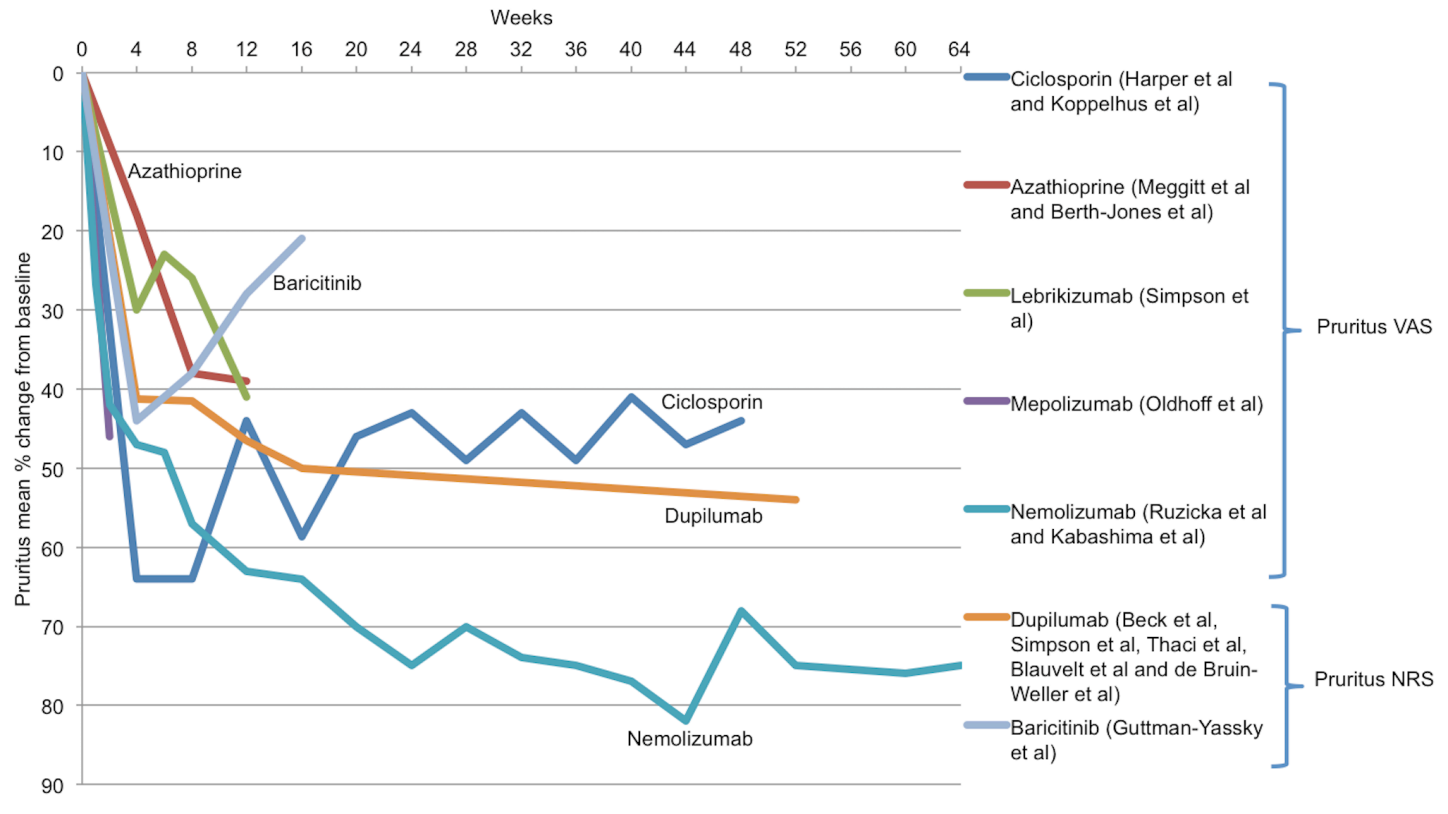
Treatment effectiveness: patient-reported severity measures. Mean percentage change from baseline in pruritus visual analogue score (VAS) and pruritus numerical rating scale (NRS) for systemic agents for which these data were reported.

A wide range of patient-assessed measures was reported across the included trials ( Supplementary Figure 3). Nemolizumab and dupilumab appear to be superior in improving pruritus compared to the other treatments ( Figure 4), although the available pruritus score data are very limited, in particular for conventional systemic agents.

### Treatment effectiveness: quality of life

**Figure 5.**
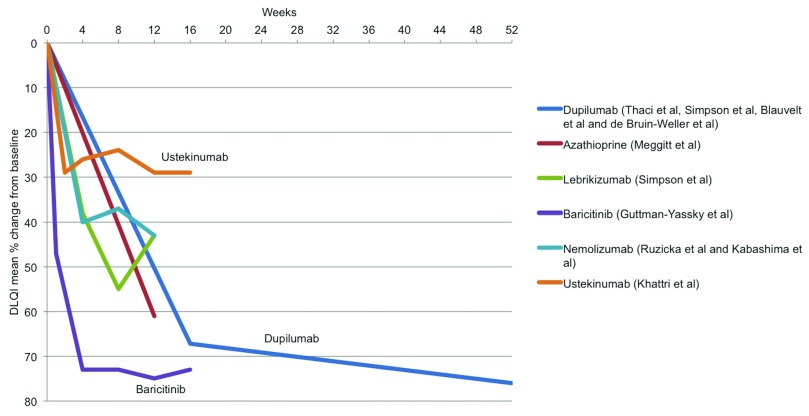
Treatment effectiveness: quality of life. Dermatology Life Quality Index (DLQI) mean percentage change from baseline for systemic agents for which these data were reported.

The Dermatology Life Quality Index (DLQI) was the most widely reported measure of quality of life, although there were several other scores reported across the trials ( Supplementary Figure 4). Baricitinib and dupilumab appear to perform best at improving quality of life ( Figure 5), although DLQI was not reported in most of the conventional systemic trials, thus precluding many of these treatments from the comparison.

### Treatment effectiveness: long-term disease control

**Figure 6.**
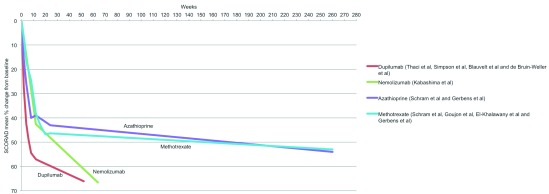
Treatment effectiveness: long-term disease control. Scoring Atopic Dermatitis (SCORAD) mean percentage change from baseline for systemic agents for which these data were reported in at least one trial that was one year or longer in duration.

Regarding long-term disease control, there are very few data available on the performance of conventional systemics and no data beyond 64 weeks for the novel systemic agents. Dupilumab and nemolizumab appear to be superior to other treatments up to one year ( Figure 6). Haeck *et al.* showed that mycophenolate sodium is also effective at maintaining remission for up to one year, after remission was induced with a six-week course of ciclosporin ^52^. A five-year follow up study comparing methotrexate and azathioprine was recently published and suggests good long-term effectiveness for both, but patient numbers in each study arm were small ^14^. The extension study SOLO-CONTINUE (NCT02395133) will provide even longer-term effectiveness data for dupilumab. Importantly, data on clinical effectiveness in terms of inducing and maintaining disease remission off treatment are lacking for any systemic therapy, although anecdotally this has been seen for methotrexate and azathioprine in particular ^14– 16^.

## Drug safety profiles

### Short-term safety

To compare the tolerability and safety of AD systemic treatments, we have calculated the incidence rates per participant per week for AEs and serious AEs (SAEs), as these were defined in individual trials (n = 26). Seven trials either did not provide any AE data or did not provide the data in a form that enabled incidence rate calculation. This highlights the lack of standardisation in safety reporting in clinical trials, limiting robust comparisons across trials. Table 2 summarises the available safety data from the included studies, showing high variability in the incidence rates reported for the conventional systemic agents. This is most likely due to the small study size and short duration of the included studies. If participant numbers are small in a RCT, then there may be misleadingly low AE reporting, falsely suggesting a more favourable drug safety profile than is seen in clinical practice.

**Table 2.** Summary of adverse events.

Systemic agent	SAE incidence rate per patient week (%)	AE incidence rate per patient week (%)	Common AEs (clinical trial incidence of ≥1/100)
Ciclosporin	0–2.2	0–20.8	Serum creatinine increase, hypertension, GI upset, infections, skin infections, headache, fatigue, cramps, paraesthesia, lower limb oedema, hypertrichosis, gingival hyperplasia, anaemia, leukopenia, pancytopenia, thrombocytopenia, ESR increase, liver enzyme increase, magnesium decrease, fever, malaise, AD exacerbation, dyslipidaemia, tremor, flushing, metallic taste
Methotrexate	0.19	9.8–23.5	GI upset, infections, liver enzyme increase, skin infections, AD exacerbation, anaemia, leukopenia, pancytopenia, fatigue, headache, renal impairment, fever, malaise
Azathioprine	0.03	3–22.9	GI upset, URTI, LRTI, fatigue, light-headedness, malaise, headache, folliculitis, skin infections, lymphopenia, neutropenia, liver enzyme increase, AD exacerbation
Mycophenolate mofetil	0	4.2	Nausea, headache, fatigue, paraesthesia, muscle ache, infections, serum creatinine increase, leukopenia, liver enzyme increase, magnesium decrease
Dupilumab	0–0.55	6.4–21.6	Nasopharyngitis, headache, URTI, injection site reactions, conjunctivitis, AD exacerbation, skin infections, herpes viral infections
Nemolizumab	0.18	6.6	Nasopharyngitis, AD exacerbation, serum CK increase, URTI, headache, peripheral oedema, impetigo, injection-site reactions
Ustekinumab	0	2.3–2.4	Nasopharyngitis, AD exacerbation
Fezakinumab	0.42	2.25	Viral URTI
Lebrikizumab	Not reported (3.2% of patients had ≥1 SAE over 20-week study)	Not reported (67% of patients had ≥1 AE over 20-week study)	Infections, skin infections, HSV and HZV infections, conjunctivitis, injection site reactions
Baricitinib	0.08%	Not reported (59% of patients had ≥1 AE over 16-week study)	Headache, serum CK increase, AD exacerbation, nasopharyngitis, cellulitis, infections
Tralokinumab	Not reported (3.3% of patients had ≥1 SAE over 12-week study)	Not reported (66% of patients had ≥1 AE over 12-week study)	Nasopharyngitis, URTI, headache, AD exacerbation, injection site reactions, arthralgia, syncope

AD, atopic dermatitis; AE, adverse event; CK, creatine kinase; ESR, erythrocyte sedimentation rate; GI, gastrointestinal; HSV, herpes simplex virus; HZV, herpes zoster virus; LRTI, lower respiratory tract infection; SAE, serious adverse event; URTI, upper respiratory tract infection.

### Long-term safety

Although pre-clinical data suggest that novel systemics may be safer than conventional agents, data on the long-term safety of both systemics in AD populations are limited ^53^. Although most cases of immunosuppression-related malignancy occur in the context of organ transplantation, studies of patients with inflammatory bowel disease have found an increased risk of lymphoma and non-melanoma skin cancer in those treated with azathioprine ^19– 22^. Furthermore, there are cases of methotrexate-associated lymphoproliferative disorders, which resolve after withdrawal of the drug, seen in adults with autoimmune diseases, such as rheumatoid arthritis ^54, 55^. However, these patients have different co-morbidities and take additional medications to those taken by AD patients, which may play a role in these associations. Cases of malignancy have been reported in RCTs of JAK inhibitors in patients with rheumatoid arthritis, and additional data from long-term follow up of patient cohorts is needed to determine the true malignancy risk for JAK inhibitors in AD ^56^.

## Cost-effectiveness

Despite novel systemic agents’ promising clinical effectiveness, there is no guarantee that they will be affordable for use within collectively funded healthcare systems. Given that innovations in medicine often impose additional costs when compared to usual care, many healthcare systems around the world now require robust evidence demonstrating that a drug therapy is cost-effective to receive approval for use in clinical practice ^57, 58^. As such, it is important to consider the evidence needed to develop a cost-effectiveness analysis when formulating data collection strategies for the evaluation of novel systemic treatments. In many countries—including England, Canada, and Australia—cost-effectiveness studies quantify changes in patient health, using the quality-adjusted life year (QALY), which combines mortality and morbidity effects in a single outcome measure ^59– 61^. Morbidity effects are typically captured using a preference-based measure of health-related quality of life (HRQoL), such as the EQ-5D instrument ^62^. In addition, data on healthcare resource use and unit costs are needed to calculate the expected healthcare costs associated with the alternative treatments being compared.

A recent systematic review by McManus *et al.* identified 24 cost-effectiveness studies evaluating interventions for the treatment and prevention of AD ^63^. One of the key findings in this review was the observed variability in the methods used to combine different forms of evidence (e.g. response rates, HRQoL effects, and resource use), also known as models. In other disease areas, such as rheumatoid arthritis and psoriatic arthritis ^64^, efforts have been made to reach a consensus on the preferred modelling approach and, as a consequence, standardise the evidence requirements. Similar efforts to establish a preferred modelling approach in the context of AD not only would provide a benchmark against which studies could be assessed but also could be used to inform future data collection strategies.

Another important finding of the systematic review was that the time horizons under examination in many of the studies were of insufficient duration. Ideally, the time horizon for any given cost-effectiveness study should reflect the length of time over which the expected costs and QALYs are likely to differ between alternative treatment strategies ^65^. Although a challenging proposition given that the follow up of patients even in observational studies continues for only three to five years, the recent appraisal of dupilumab by NICE demonstrates that observational evidence can be used in addition to RCT data to inform the cost-effectiveness analysis of patients ^66^. Partly based on this health economic analysis, NICE has recommended dupilumab as an option for treating moderate-to-severe AD in adults where the disease has not responded to at least one conventional systemic therapy or where these are contraindicated or not tolerated ^66^. Interestingly, the economic model considered as part of the NICE appraisal compared dupilumab with topical therapy as best supportive care. The rationale for this was that dupilumab would be positioned after conventional systemics in the treatment pathway for AD, at which point the only other treatment option for patients would be topical therapy ^67^. Topical therapy might indeed be considered best supportive care once a patient has failed on *all* conventional systemics; however, this was not the population of patients studied in the dupilumab trials. Indeed, only one trial, LIBERTY-CAFÉ, required participants to have a prior history of ciclosporin use (or contraindication). It is likely that the effectiveness of topical treatment varies in different populations and would be expected to be lower in a population of patients with AD who have failed multiple systemic treatments compared to those who are systemic treatment naïve. A cost-effectiveness model for novel agents should therefore be compared to conventional systemic treatment as best supportive care, even if this is challenging given the current lack of data on the cost-effectiveness of conventional systemics.

The formation of the UK–Irish Atopic eczema Systemic Therapy Register (A*STAR) and other national AD registers within the international TREatment of ATopic eczema registry taskforce (TREAT) represents an important milestone in the collection of data that can be used to inform cost-effectiveness studies for the evaluation of novel AD therapies ^68, 69^. These treatment registers will be an important data source for the development of future appraisal submissions to regulatory bodies, such as NICE in the UK, given that they collect longitudinal disease severity, quality of life, and healthcare resource use data for every patient recruited and compare conventional and novel therapies alike.

## Indirect comparison method limitations

The indirect comparison of clinical effectiveness, safety data, and cost-effectiveness drawn from different studies, as presented here, has important limitations. Such comparisons lose the benefits derived from randomisation, such as the balancing of effect modifiers across treatment groups at baseline. Whilst direct comparisons from individual studies may have a low risk of bias (i.e. high internal validity), the inferences drawn from comparisons between studies must be considered observational data and are therefore at higher risk of bias. For example, if the baseline characteristics of the trial participants, such as disease severity, differ, then it is difficult to compare a change in disease severity across trials even where the same disease severity measure was used. Other study design differences will additionally impact on treatment effectiveness, such as the use of concomitant topical corticosteroids.

## Where do we go from here?

Further head-to-head active comparator studies are needed, such as the ongoing TREatment of severe Atopic eczema Trial (TREAT), which compares ciclosporin and methotrexate in children with moderate-to-severe AD ^70^. Such studies need to use validated outcome measures that are harmonised to allow for comparisons to be made across studies. The Harmonising Outcome Measures for Eczema (HOME) initiative is an international effort towards a core outcome set for clinical trials in AD that will help to facilitate this ^71^. Whilst not a current aim of the HOME initiative, reporting would also be useful.

Recognising that randomised controlled trial data directly comparing active treatments will always be limited, network meta-analysis (NMA) will be helpful in plugging this evidence gap. NMA is an approach whereby direct and indirect data can be combined into a single statistical model that takes into account variations between studies and where the assumptions around the similarities between studies can be evaluated ^72^.

Although novel systemic agents may not vastly improve the clinical effectiveness outcomes in AD, they have the potential to address the more pressing clinical issue of short- and long-term AEs secondary to AD treatment. The limited duration of clinical trials, relatively small sample sizes with respect to safety outcomes, and issues around the generalisability of their findings to the wider patient population mean that data from large ‘real-world’ studies, such as registers and electronic health records, will play an important role in providing evidence to support clinical decision-making. Standardised AE reporting is also needed as we approach an era where safety and tolerability may become more of a differentiator of treatments than clinical effectiveness.

## Conclusion

Novel biologic and small molecule agents provide great promise for patients with moderate-to-severe AD. While the clinical effectiveness data from these placebo-controlled RCTs are promising, important factors in addition to short- and long-term treatment effectiveness need to be considered, such as drug safety and cost-effectiveness as well as the ability of a drug to alter the natural history of AD. While evidence generation through head-to-head trials would be the preferred route, such trials are likely to remain low in number. Treatment registers and NMA provide important additional tools to better inform evidence-based treatment decisions made by physicians, patients, and regulatory bodies alike. We have entered an exciting era in AD therapeutics but need to travel a much longer journey before the promise of a therapeutic revolution becomes a reality.

## Abbreviations

AD, atopic dermatitis; AE, adverse event; DLQI, Dermatology Life Quality Index; EASI, Eczema Area and Severity Index; HOME, Harmonising Outcome Measures for Eczema; HRQoL, health-related quality of life; IL, interleukin; JAK, Janus kinase; NICE, National Institute for Health and Care Excellence; NMA, network meta-analysis; QALY, quality-adjusted life year; RCT, randomised controlled trial; SAE, serious adverse event; SCORAD, Scoring Atopic Dermatitis.

## Supplementary material

**Supplementary Figure 1. Physician-assessed severity score end points of included trials.**

Different clinical severity score endpoints were reported across included randomised controlled trials. Many trials did not report the same scores, making indirect comparison of some treatments very difficult. EASI, Eczema Area and Severity Index; IGA, Investigator’s Global Assessment; SASSAD, Six Area Six Sign Atopic Dermatitis; SCORAD, Scoring Atopic Dermatitis.

**Supplementary Figure 2. Treatment effectiveness: physician-assessed severity measures.**

Eczema Area and Severity Index (EASI) (a) and Scoring Atopic Dermatitis (SCORAD) (b) mean percentage change from baseline up to 64 weeks for systemic agents for which these data were reported. MTX, methotrexate.

**Supplementary Figure 3. Treatment effectiveness: patient-reported severity measures.**

The number of included trials reporting patient-reported severity measures. HADS, Hospital Anxiety and Depression Scale; NRS, numerical rating scale; POEM, Patient Oriented Eczema Measure; RCT, randomised controlled trial; SCORAD, Scoring Atopic Dermatitis; VAS, visual analogue scale.

**Supplementary Figure 4. Treatment effectiveness: quality of life.**

The number of included trials reporting quality of life measures. AD, atopic dermatitis; cDLQI, children’s Dermatology Life Quality Index; DLQI, Dermatology Life Quality Index; RCT, randomised controlled trial; VAS, visual analogue scale.
