# Supplementary figures and images for: Novel systemic therapies in atopic dermatitis: what do we need to fulfil the promise of a treatment revolution?
Source: F1000Res. 2019 Jan 31;8:F1000 Faculty Rev-132. [Version 1] doi: 10.12688/f1000research.17039.1 (PMC6357995; doi:10.12688/f1000research.17039.1)

Figure 1

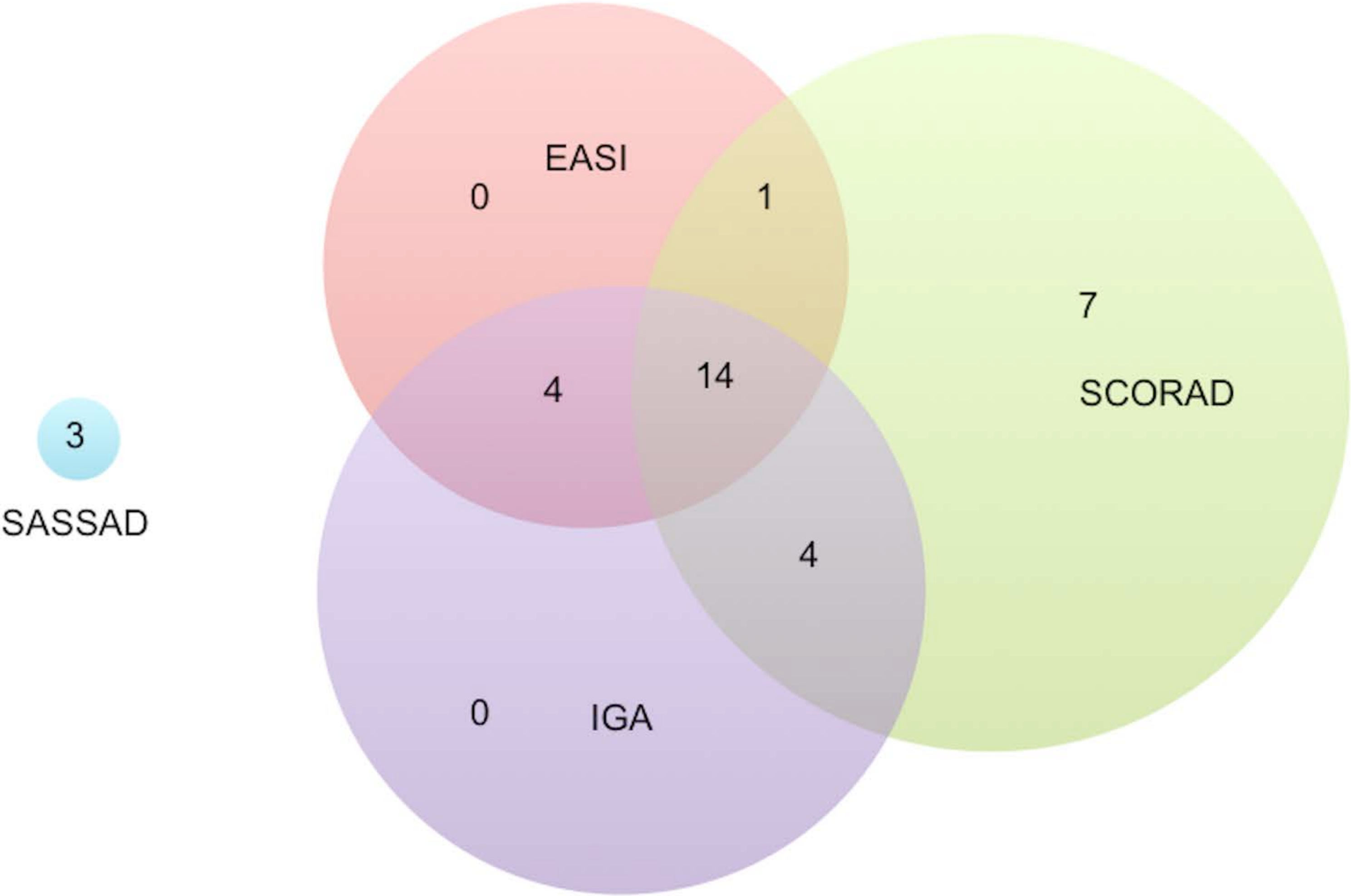

Supplement: Supplementary file 1 [file f1000research-8-18628-s0000.tgz › b3c1735d-d997-4e03-8704-eac91a1032c5_Suppl_fig1.pdf]

Figure 2A

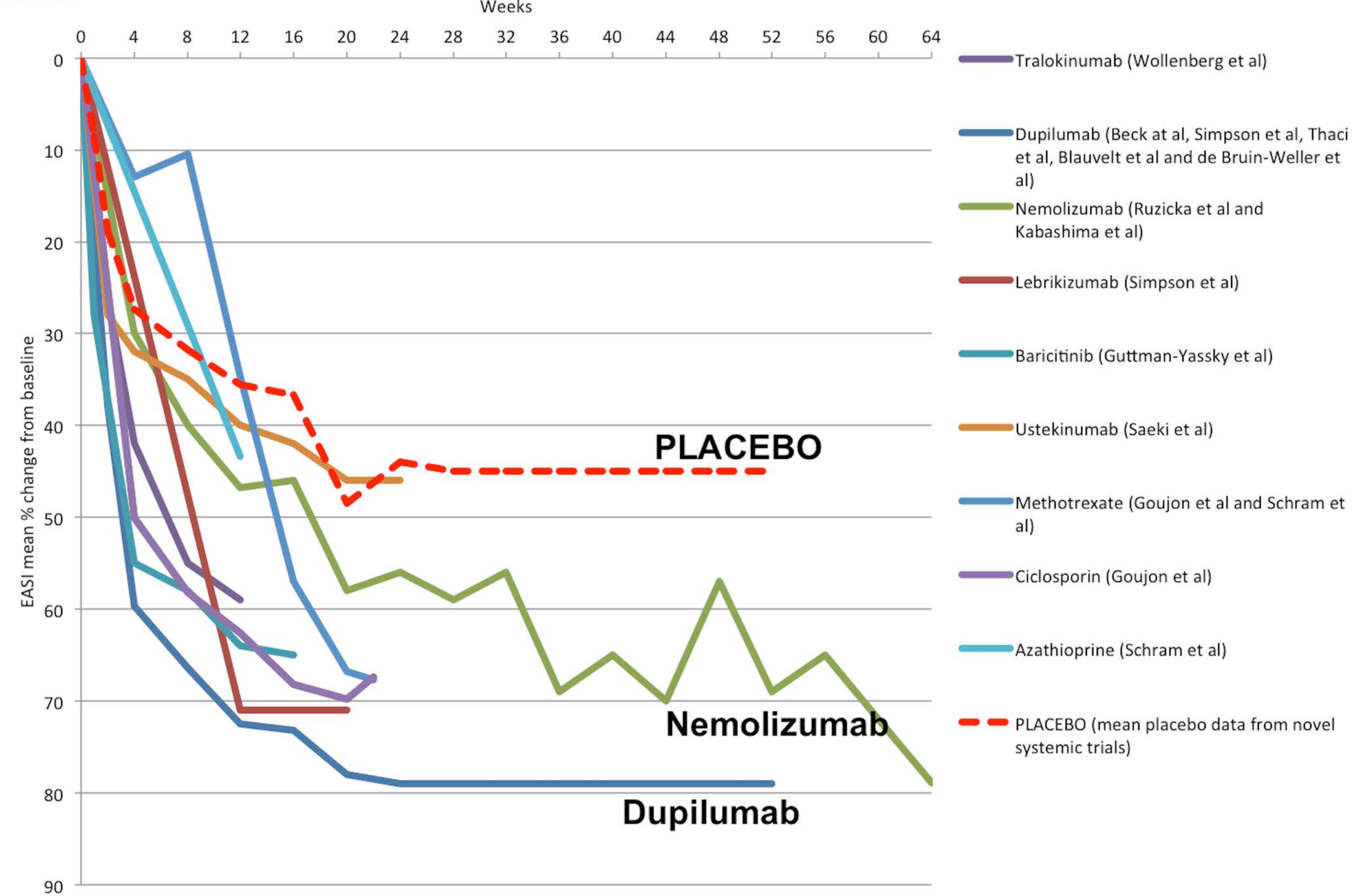

Figure 2B

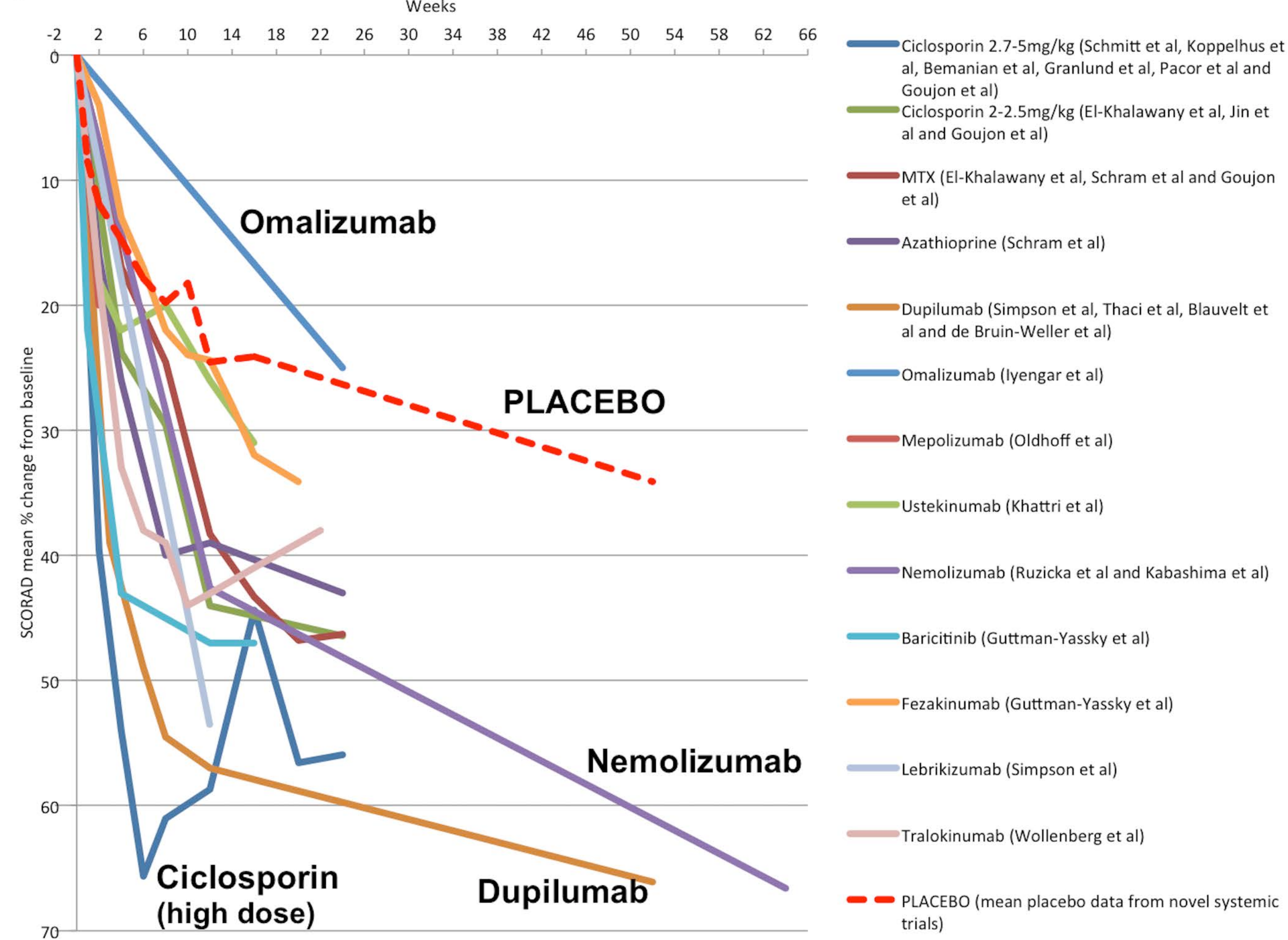

Supplement: Supplementary file 2 [file f1000research-8-18628-s0001.tgz › 857ca55e-89e8-486b-850a-c434b3612dce_Suppl_fig2.pdf]

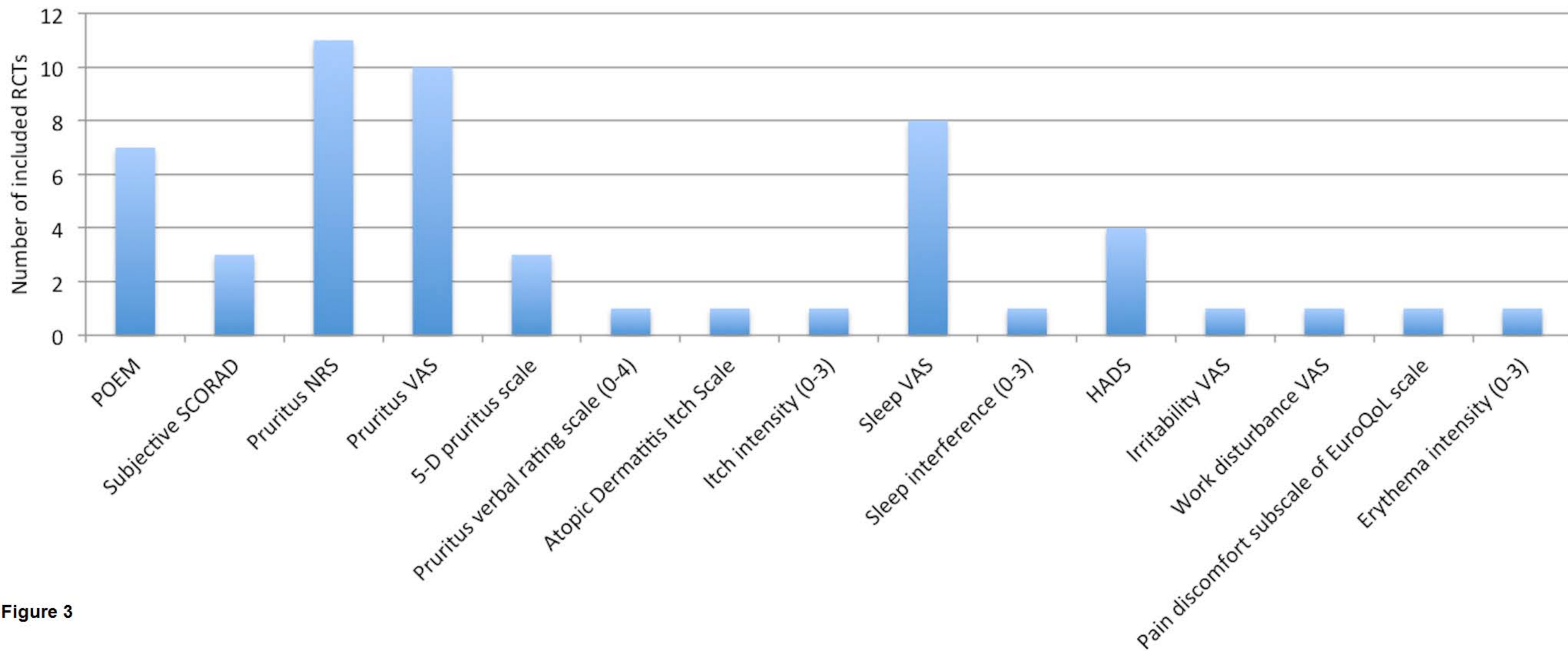

Figure 3

Supplement: Supplementary file 3 [file f1000research-8-18628-s0002.tgz › 801a2615-fa15-4c8b-a53a-9c978351de48_Suppl_fig3.pdf]

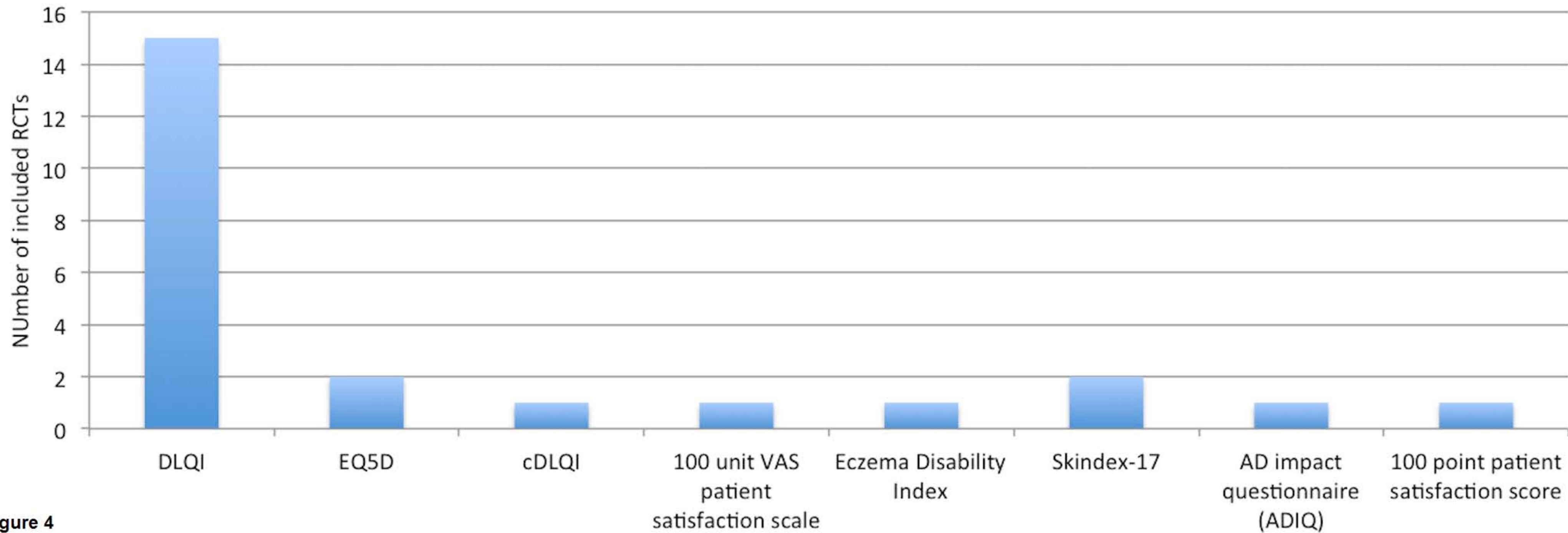

Figure 4

Supplement: Supplementary file 4 [file f1000research-8-18628-s0003.tgz › 2db742ad-a58d-4c66-a1fe-124d8899b56f_Suppl_fig4.pdf]
